# Supplementary material for: The heritability of multi-modal connectivity in human brain activity
Source: eLife. 2017 Jul 26;6:e20178. doi: 10.7554/eLife.20178 (PMC5621837; doi:10.7554/eLife.20178)
Supplement: Supplementary file 1. [file elife-20178-supp1.pdf]

| ROI number | ROI location |
|---:|---|
| 1, 2, 3, 4, 5 | Left Frontal Lobe |
| 6 | Left Somatosensory Cortex |
| 7, 8 | Left Motor Cortex |
| 9, 10, 11 | Left Parietal Cortex |
| 12, 13 | Left Visual Cortex |
| 14, 15 | Left Occipital Lobe |
| 16, 17, 18 | Left Temporal Lobe |
| 19, 20, 21 | Right Temporal Lobe |
| 22, 23 | Right Occipital Lobe |
| 24, 25 | Right Visual Cortex |
| 26, 27, 28, 29, 30 | Right Parietal Lobe |
| 31 | Right Motor Cortex |
| 32 | Right Somatosensory Cortex |
| 33, 34, 35, 36, 37 | Right Frontal Lobe |
| 38 | Posterior Cingulate Cortex |
| 39 | Medial Frontal Cortex |

Table 1: Index of ROI numbers.
