# Supplementary material for: The heritability of multi-modal connectivity in human brain activity
Source: eLife. 2017 Jul 26;6:e20178. doi: 10.7554/eLife.20178 (PMC5621837; doi:10.7554/eLife.20178)
Supplement: Supplementary file 2. [file elife-20178-supp2.pdf]

	Estimated parameter	value	95% confidence
Connectivity ACE model (θ)	h^2	0.07	$[0.00, 0.27]$
	c^2	0.06	$[0.00, 0.38]$
	$h^2 - c^2$	0.01	$[-0.42, 0.37]$
Connectivity ACE model (α)	h^2	0.08	$[0.01, 0.25]$
	c^2	0.06	$[0.00, 0.20]$
	$h^2 - c^2$	0.03	$[-0.66, 0.34]$
Connectivity ACE model (β)	h^2	0.19	$[0.13, 0.26]$
	c^2	0.06	$[0.00, 0.12]$
	$h^2 - c^2$	0.13	$[0.04, 0.22]$
Connectivity ACE model (fMRI)	h^2	0.17	$[0.15, 0.18]$
	c^2	0.02	$[0.01, 0.03]$
	$h^2 - c^2$	0.15	$[0.13, 0.16]$
Power ACE model (θ)	h^2	0.07	$[0, 0.41]$
	c^2	0.13	$[0, 0.49]$
Power ACE model (α)	h^2	0.06	$[0, 0.36]$
	c^2	0.06	$[0, 0.38]$
Power ACE model (β)	h^2	0.04	$[0, 0.38]$
	c^2	0.05	$[0, 0.36]$
Power ACE model (fMRI)	h^2	0.35	$[0.30, 0.41]$
	c^2	0.02	$[0, 0.04]$

Table 2: Parameter estimates and 95% confidence intervals for the mean genetic and shared environmental contributions to the observed phenotypic variability in functional connectivity and signal power, using the 39-dimensional parcellation derived from a high-dimensional ICA on fMRI data.
