# Supplementary material for: The heritability of multi-modal connectivity in human brain activity
Source: eLife. 2017 Jul 26;6:e20178. doi: 10.7554/eLife.20178 (PMC5621837; doi:10.7554/eLife.20178)
Supplement: Supplementary file 3. [file elife-20178-supp3.pdf]

	Test	p	p_{FDR}
Network Dissimilarity (θ)	$UN > DZ$	$< 10^{-4}$	$< 10^{-4}$
	$DZ > MZ$	3×10^{-4}	7×10^{-4}
Network Dissimilarity (α)	$UN > DZ$	$< 10^{-4}$	$< 10^{-4}$
	$DZ > MZ$	3×10^{-3}	6×10^{-3}
Network Dissimilarity (β)	$UN > DZ$	$< 10^{-4}$	$< 10^{-4}$
	$DZ > MZ$	$< 10^{-4}$	$< 10^{-4}$
Network Dissimilarity (fMRI)	$UN > SI$	$< 10^{-4}$	$< 10^{-4}$
	$SI > DZ$	$< 10^{-4}$	$< 10^{-4}$
	$DZ > MZ$	$< 10^{-4}$	$< 10^{-4}$
Connectivity ACE model (θ)	$h^2 > 0$	0.35	0.39
Connectivity ACE model (α)	$h^2 > 0$	0.02	0.03
Connectivity ACE model (β)	$h^2 > 0$	6.0×10^{-3}	0.01
Connectivity ACE model ($fMRI$)	$h^2 > 0$	1.0×10^{-4}	3×10^{-4}
Power ACE model (θ)	$h^2 > 0$	0.28	0.35
Power ACE model (α)	$h^2 > 0$	0.28	0.35
Power ACE model (β)	$h^2 > 0$	0.34	0.39
Power ACE model (fMRI)	$h^2 > 0$	0.02	0.03

Table 3: p-values for permutation-based significance tests performed for the strength of genetic factors, both before and after a false discovery rate correction for multiple comparisons over the 21 tests performed in this article.
