# Supplementary material for: The heritability of multi-modal connectivity in human brain activity
Source: eLife. 2017 Jul 26;6:e20178. doi: 10.7554/eLife.20178 (PMC5621837; doi:10.7554/eLife.20178)
Supplement: Supplementary file 4. [file elife-20178-supp4.pdf]

	Estimated parameter	value	95% confidence
Connectivity ACE model (θ)	h^2	0.05	$[0.00, 0.10]$
	c^2	0.08	$[0.04, 0.13]$
	$h^2 - c^2$	-0.03	$[-0.12, 0.03]$
Connectivity ACE model (α)	h^2	0.14	$[0.08, 0.22]$
	c^2	0.11	$[0.05, 0.17]$
	$h^2 - c^2$	0.03	$[0.00, 0.20]$
Connectivity ACE model (β)	h^2	0.14	$[0.08, 0.22]$
	c^2	0.11	$[0.05, 0.17]$
	$h^2 - c^2$	0.03	$[-0.06, 0.14]$
Connectivity ACE model (fMRI)	h^2	0.29	$[0.26, 0.33]$
	c^2	0.03	$[0.01, 0.04]$
	$h^2 - c^2$	0.27	$[0.22, 0.31]$

Table 4: Parameter estimates and 95% confidence intervals for the mean genetic and shared environmental contributions to the observed phenotypic variability in functional connectivity and signal power, using the 15-dimensional ICA parcellation from fMRI data.
