# Supplementary material for: The heritability of multi-modal connectivity in human brain activity
Source: eLife. 2017 Jul 26;6:e20178. doi: 10.7554/eLife.20178 (PMC5621837; doi:10.7554/eLife.20178)
Supplement: Supplementary file 5. [file elife-20178-supp5.pdf]

	ρ	p	p_{FDR}
fMRI	-0.57	1.00	1.00
Beta-band	-0.04	0.60	0.63
Alpha-band	0.15	0.18	0.25
Theta-band	0.39	0.01	0.02

Table 5: Correlations over ROIs, with permutation-based p-values, between the average heritability of cortical curvature in each ROI and the average heritability of connections from each ROI. A lack of strong positive correlations suggests that any heritability in cortical curvature is not driving the heritability observed in functional connection strengths. p-values are given both uncorrected, and after a false discovery rate correction for multiple comparisons over the 21 tests performed in this article.
